# A priority health index identifies the top six priority risk and related factors for non-communicable diseases in Brazilian cities

**DOI:** 10.1186/s12889-015-1787-1

**Published:** 2015-05-01

**Authors:** Eduardo J Simoes, Adam Bouras, Juan Jose Cortez-Escalante, Deborah C Malta, Denise Lopes Porto, Ali H Mokdad, Lenildo de Moura, Otaliba Libanio Morais Neto

**Affiliations:** Department of Health Management and Informatics, Universityof Missouri School of Medicine, Columbia, USA; Min. Saude, Secret. de Vigil.Saude, Depart. de Vigilancia de Doencas e Agravos Nao Transmiss. & Promocao de Saude, Coord. Geral de Inform. e Analise Epidemiologica, Brasilia, Brazil; Institute for Health Metrics and Evaluation, University of Washington, Seattle, USA; Organizacao Pan-Americana de Saude, Brasilia,, Brazil; Instituto de Patologia Tropical e Saúde Pública, Universidade Federal de Goiás, Goiania, Brazil

## Abstract

**Background:**

In Brazil, 72% of all deaths in 2007 were attributable to non-communicable diseases (NCD). We used a risk and related factor based index to prioritize NCD prevention programs in the combined 26 capital cities and the federal district (i.e., Brasilia) of Brazil.

**Methods:**

We used 2006–2011 data (adults) from census and Brazil's surveillance of 12 NCD risk factors and 74 disease group mortality. The risk and related factors were: smoking, physical inactivity, overweight-obesity, low fruits and vegetables intake, binge drinking, insufficient Pap smear screening (women aged 25 to 59 years), insufficient mammography screening (women aged 50 to 69 years), insufficient blood pressure screening, insufficient blood glucose screening, diagnosis of hypercholesterolemia, diagnosis of hypertension and diagnosis of diabetes. We generated six indicators: intervention reduction of the risk factor prevalence, intervention cost per person, prevalence of risk factor, deaths attributable to risk factor, risk factor prevalence trend and ratio of risk factor prevalence between people with and without a high school education. We transformed risk and related factor indicators into priority scores to compute a priority health index (PHI). We implemented sensitivity analysis of PHI by computing it with slightly altered formulas and altering values of indicators under the assumption of bias in their estimation. We ranked risk factors based on PHI values.

**Results:**

We found one intermediate (i.e., overweight-obesity) and six top risk and related factors priorities for NCD prevention in Brazil's large urban areas: diagnosed hypertension, physical inactivity, blood pressure screening, diagnosed hypercholesterolemia, smoking and binge drinking.

**Conclusion:**

Brazil has already prioritized the six top priorities (i.e., hypertension, physical inactivity, blood pressure screening, hypercholesterolemia, smoking and binge drinking) and one intermediate priority (i.e., overweight-obesity) for NCD prevention identified in this report. Because effective interventions to reduce disease burden associated with each of the six priority risk factors are available, strategies based on these interventions need to be sustained in order to reduce NCD burden in Brazil. PHI can be used to track NCD prevention and health promotion actions at the local and national level in Brazil and in countries with similar public health surveillance systems.

**Electronic supplementary material:**

The online version of this article (doi:10.1186/s12889-015-1787-1) contains supplementary material, which is available to authorized users.

## Background

In Brazil, 72% of all deaths in 2007 were attributable to non-communicable diseases (NCDs) [1]. As Brazil’s population continued to grow and age [2-4], the burden of NCDs will increase at a time when the country is still dealing with a threat of infectious diseases and poverty-related health outcomes [4,5].

NCD prevention and health promotion policies and strategies of Brazil, especially at city level, have been strengthened after enactment in 1990 of the 1988 federal health reform and the creation of Brazil’s Universal Health Care System (SUS) that covers the Brazilians for free [6]. This decentralized system is directly run by each municipality and funded by a national health fund.

As of 2010, approximately 86% of Brazil’s populations lived in an urban center (i.e., city and vicinity) with about 32% living in 16 major metropolitan regions and 42% living in 26 state capital cities and the city of Brasilia (i.e., federal district of Brazil) [7]. This level of urbanization and associated lifestyle changes (e.g., compressed leisure time, smoking, poor diet, increased alcohol consumption) contributes to increases in NCD and poor population health in Brazil [8,9].

Because funds are limited, public health programs need to be prioritized [10,11]. Hence, we used the priority health index (PHI), a prioritization methodology applied in the State of Missouri in the United States since 2000 [12,13] and Italy [14] to prioritize public health programs for NCD in the combined 26 state capital cities and the city of Brasilia. More specifically, our study objectives were to: 1) use Brazil’s public health surveillance data to generate NCD focused PHI; 2) use PHI to identify NCD priorities for 27 capital cities in Brazil; and 3) compare the PHI identified priorities with prevention priorities in Brazil.

## Methods

PHI utilizes readily available public health surveillance data to prioritize health issues by balancing the relative impact of indicators across six criteria of prioritization.

We used all available data from 2000 to 2011 from the Brazil's Surveillance System on Risk and Related Factors (hereafter referred to as factor) for Non-transmissible Disease through Telephone Interviews (VIGITEL) [15,16], Information System for Mortality (SIM) [17], and census data [18] in all 27 cities. VIGITEL, launched in 2006, provided continuous surveillance data based on about 54,000 completed interviews with adults per year (around 2,000 per state capital) [15]. SIM captures all causes of death, location of death, residency location and socio-demographics of the deceased individuals [17]. We focused on 12 NCD factors and 74 groups of diseases and health conditions to calculate PHI.

The 12 NCD factors defined by VIGITEL were (Appendix 1): being a current smoker (smoking); being sedentary or reaching insufficient levels of physical activity (physical inactivity); having a body mass index (BMI) greater than 25 (overweight- obesity); consuming fruits and vegetables fewer than five times in a week (low fruits and vegetables intake); consuming more than five standard alcoholic drinks on a single occasion for men (four doses for women) (binge drinking); women (25–59 years) who have not had a Pap test in the last three years (insufficient Pap smear screening); women (50–69 years) who have not had a mammography in the last two years (insufficient mammography screening); having not had a blood pressure measured in the last one year (insufficient BP screening); having not had a blood glucose measured in the last two years (insufficient glucose screening); having been diagnosed with hypercholesterolemia (hypercholesterolemia); having been diagnosed with hypertension (hypertension); and having been diagnosed with diabetes (diabetes) [15].

We generated six indicators within the criteria: intervention effectiveness (relative reduction in the prevalence of a risk factor); intervention cost (cost per person reached by an effective intervention), magnitude (prevalence of the risk factors); severity (deaths attributable to the risk factors); urgency (risk factor prevalence trend over time); disparity (ratio of risk factor prevalence between low to high education attainment). PHI modulates the population health burden measured by the number of deaths attributable to a factor with the inclusion of other priority criteria: magnitude and urgency of the risk factor and its presented socioeconomic disparity; the cost and effectiveness of an intervention to reduce the risk factor magnitude and disparity, and stem its rate of increase [14].

We used the 2011 prevalence of a factor, relative risks for the relationship between risk factors, and mortality by age-sex specific groups to calculate the mortality attributable to each risk factor. The underlying causes of deaths by ICD 9 and ICD 10 for the 75 diseases or conditions by specific age-sex groups are presented in Appendix 2. We used relative risks previously published [19]. We then generated age and gender specific population attributable fractions (PAF) (Appendix with spreadsheet of calculations is available upon request). We used the following formula: PAF = (P_0_ + P_1_RR_1_ + P_2_RR_2_ + … + P_K_RR_K_) -1/(P_0_ + P_1_RR_1_ + P_2_RR_2_ + … + P_K_RR_K_) to adjust for levels of risk factors [20]. These risk factors were: cigarette smoking (never smokers, former smokers, current smokers); alcohol consumption (males: abstainers, 0–39 g, 40–59 g, 60+ g, and binge; females: abstainers, 0–19 g, 20–39 g, 40+ g and binge); and physical inactivity (highly active, active, insufficient, inactive). All other nine risk factors were dichotomous (yes/no). We used the following formula to estimate PAF for dichotomous variables: PAF = P(RR-1)/1+ (RR-1) [21]. We then multiplied PAF by death counts to generate population attributable deaths (severity criterion).

The urgency criterion is delta (Δ), the coefficient of linear trend of the prevalence of the risk factor between 2006 and 2011. We estimated Δ by the slope coefficient (i.e., β1) of the regression line for the period of time: Y = β0 + β1 X; where Y = the prevalence of the risk factor and X = time in year. We set the Δ value to zero to indicate no trend if the regression coefficient is not statistically significant (using two-sided test with p-values ≤0.05). Hence, if the prevalence is increasing over time, it indicates that this risk factor is more urgent than that of another risk factor for which the prevalence is decreasing or stable. Because diabetes and blood pressure screening were only available for 2010 and 2011 through VIGITEL, we estimated their delta using the following formula: Δ = (P_f_ / P_b_ -1)/n-1; where n is the number of years, P_f_ and P_b_ are the prevalence in the last and preceding years, respectively.

We defined socio-economic disparity in health as the 2011 risk factor prevalence ratio between persons with fewer than 12 years of education and 12 or more years of education. We used the value of the ratio when statistically significant (using two-sided test with p-values ≤0.05) or greater than 10%; otherwise, a value of 1 was assigned.

We used intervention effectiveness and cost measures from a review of the English literature between 1990 and 2009 m. Intervention cost and effectiveness measures for the prevention of chronic diseases were unavailable in the Portuguese literature. We used the following formula to estimate effectiveness: Effect = P _final_ – P _baseline_, where P _final_ is the prevalence at the end of the intervention follow-up period and P _baseline_ is the prevalence at the beginning of the follow-up period. We used the per capita cost of a public health intervention for the duration of the intervention study to estimate the cost criterion. Because cost and effectiveness data were unavailable for diabetes screening, we used the available cost and effectiveness measures for cholesterol screening as proxy.

The magnitude of the risk factors was estimated by the prevalence of the risk factor in 2011.

We standardized all our risk factor indicators to generate dimensionless and comparable scores. We re-scaled our standardized scores to avoid negative values. We used the following formula to transform the indicator into a re–scaled and standardized score: S = 3 + ( I – Avg (I) )/Sd (I), where S is the score of the indicator (I), 3 is a re-scale constant, I is the indicator (e.g., linear coefficient on risk factor prevalence between 2006 and 2011), Avg (I) is the mean of the indicator I across all risk factors, and Sd (I) is the standard deviation of the indicator I across all risk factors. We divided the cost value by 1000 before standardizing and re-scaling it in order to keep all indicators with identical orientation in the PHI formula (i.e., higher values equal higher priority ranking). We generated three PHI as a sensitivity analysis. We created three PHI: the sum of the priority ranked values of indicators (A), product of the scores (B), and sum of scores (C) across the seven criteria. We created one composite PHI measure: the weighted average of the ranking values of a risk factor for indexes A, B and C (Composite D). We ranked the risk factors based on the PHI values.

### Ethical considerations

The data collection done by the VIGITEL and SIM were approved by the National Human Research Ethics Committee of the Brazilian Ministry of Health.

## Results

The original indicators and the score values are presented in Tables 1 and 2. The highest disparity scores were insufficient Pap smear and mammography screening, followed by diabetes. Overweight-obesity and diabetes presented the highest urgency due to recent increasing trends in prevalence, while overweight-obesity and low fruits/vegetables intake had the highest magnitude. The factors associated with the highest mortality burden per the severity score were hypertension, heavy alcohol consumption and smoking, followed by physical inactivity and insufficient BP screening. Hypercholesterolemia, diabetes, hypertension, physical inactivity, and BP screening had the highest scores for intervention effectiveness. Binge drinking, physical inactivity, smoking, hypertension, hypercholesterolemia and BP screening had the lowest cost score.

**Table 1:** **Risk factor priority indicator values: combined 27 capital cities total**

**Risk factor**	**Cost** ^**1**^	**Cost transformed** ^**2**^	**Effectiveness** ^**3**^	**Severity** ^**4**^	**Magnitude** ^**5**^	**Urgency** ^**6**^	**Disparity** ^**7**^
Current smoking	4.90	204.082	0.116	21298.000	0.150	−0.310	1.520
Physical inactivity	4.30	232.558	0.350	17505.000	0.150	0.000	0.890
Overweight-obesity	41.70	23.981	0.095	2453.000	0.480	1.390	1.000
Eating < 5 servings of fruit and vegetables per week	10.00	100.000	0.032	904.000	0.700	0.000	1.290
Abusive Alcohol Intake (binge drinking)	4.00	250.000	0.080	22927.000	0.190	0.000	0.810
Had no Papanicolaou in last three years	11.40	87.719	0.180	66.000	0.190	0.000	2.060
Had no Mammography in last two years	550.00	1.818	0.182	79.000	0.270	−0.560	2.410
Had no BP Screening in last one year	10.00	100.000	0.317	9816.000	0.210	0.000	1.600
Had no Glucose Screening in last two years	41.70	23.981	0.076	414.000	0.110	0.000	1.470
Has been diagnosed with high cholesterol	10.00	100.000	0.470	5518.000	0.170	0.000	1.000
Has been diagnosed with hypertension	10.00	100.000	0.225	25402.000	0.230	0.000	1.360
Has been diagnosed with diabetes	10.00	100.000	0.235	1071.000	0.060	0.170	1.620
Mean	-	110.345	0.197	8954.417	0.243	0.058	1.419
Standard deviation	-	80.323	0.131	10016.920	0.177	0.460	0.477

^1^Intervention Cost (US$) per Person Covered.

^2^Transformation of Intervention Cost per Person Covered (i.e., 1000/intervention cost).

^3^Percent reduction in the prevalence of Risk Factor due to Intervention.

^4^Mortality Attributable to Risk Factor.

^5^Prevalence of Risk Factor.

^6^Unit of change in Risk Factor prevalence per year (Slope).

^7^Low (<12 years) to High (= > 12 years) Education Ratio of the Risk Factor Prevalence.

**Table 2:** **Two Risk factor priority indicator scores** ^**&,!**^ **for the combined 27 cities total**

	**Cost** ^**1**^		**Effectiveness** ^**2**^		**Severity** ^**3**^		**Magnitude** ^**4**^		**Urgency** ^**5**^		**Disparity** ^**6**^	
**Risk factor**	**Score1**	**Score2**	**Score1**	**Score2**	**Score1**	**Score2**	**Score1**	**Score2**	**Score1**	**Score2**	**Score1**	**Score2**
Current smoking	4.167	3	2.384	8	4.232	3	2.479	9	2.202	11	3.211	5
Physical inactivity	4.522	2	4.175	2	3.854	4	2.479	9	2.875	3	1.890	11
Overweight-obesity	1.925	10	2.223	9	2.351	7	4.338	2	5.895	1	2.121	9
Eating < 5 servings of fruit and vegetables per week	2.871	4	1.740	12	2.196	9	5.578	1	2.875	3	2.729	8
Abusive alcohol intake (binge drinking)	4.739	1	2.108	10	4.395	2	2.704	6	2.875	3	1.722	12
Had no papanicolaou in last three years	2.718	9	2.874	7	2.113	12	2.704	6	2.875	3	4.344	2
Had no mammography in last two years	1.649	12	2.889	6	2.114	11	3.155	3	1.659	12	5.078	1
Had no BP screening in last one year	2.871	4	3.923	3	3.086	5	2.817	5	2.875	3	3.379	4
Had no Glucose Screening in last two years	1.925	10	2.077	11	2.147	10	2.253	11	2.875	3	3.107	6
Has been diagnosed with high cholesterol	2.871	4	5.094	1	2.657	6	2.591	8	2.875	3	2.121	9
Has been diagnosed with hypertension	2.871	4	3.218	5	4.642	1	2.930	4	2.875	3	2.876	7
Has been diagnosed with diabetes	2.871	4	3.295	4	2.213	8	1.972	12	3.244	2	3.421	3

&Score1 is created by re-scaling and standardizing the risk factor indicator.

^!^Score2 is the ranking value of the risk factor indicator.

^1^Intervention Cost (US$) per Person Covered.

^2^Transformation of Intervention Cost per Person Covered (i.e., 1000/intervention cost).

^3^Percent reduction in the prevalence of Risk Factor due to Intervention.

^4^Mortality Attributable to Risk Factor.

^5^Prevalence of Risk Factor.

^6^Unit of change in Risk Factor prevalence per year (Slope).

^7^Low (<12 years) to High (= > 12 years) Education Ratio of the Risk Factor Prevalence.

The priority health indices (A, B and C), and the composite priority index (D) are presented in Table 3. Hypertension, physical inactivity, and blood pressure screening were ranked at the top across priority indices A, B and C and composite index D.

**Table 3:** **Risk factor ranking by priority health indexes (with risk factors ordered from top to lowest based on the composite priority index D)**

**Risk factor**	**Priority health index A** ^**a**^	**Priority rank**	**Priority health index B** ^**b**^	**Priority rank**	**Priority health index C** ^**c**^	**Priority rank**	**Composite Index D** ^**d**^	**Priority rank**
Has been diagnosed with hypertension	24	1	1039.002	1	19.41199	2	1.33	1
Physical Inactivity	31	3	980.0549	2	19.79463	1	2.00	2
Had no BP screening in last one year	24	1	951.235	3	18.95115	3	2.33	3
Has been diagnosed with high cholesterol	31	3	614.1151	5	18.20992	7	5.00	4
Current smoking	39	9	736.7486	4	18.67477	5	6.00	5
Abusive alcohol intake (binge drinking)	34	6	587.8737	6	18.54317	6	6.00	5
Overweight-Obesity	38	8	545.5287	8	18.85233	4	6.67	7
Eating < 5 servings of fruit and vegetables per week	37	7	480.3386	9	17.99008	8	8.00	8
Had no papanicolaou in last three years	39	9	557.361	7	17.62787	9	8.33	9
Has been diagnosed with diabetes	33	5	458.1501	10	17.01621	10	8.33	10
Had no mammography in last two years	45	11	267.5791	11	16.54336	11	11.00	11
Had no glucose screening in last two years	51	12	172.8065	12	14.38453	12	12.00	12

^a^Sum of the ranking values of the risk factor indicators.

^b^Product of re-scaled and standardized risk factor Indicators.

^c^Sum of re-scaled and standardized risk factor indicators.

^d^Composite Index consisting of the average of the rankings of priority indexes a, b and c.

Hypertension, physical inactivity, blood pressure screening, diagnosed hypercholesterolemia, smoking and binge drinking were the top five ranked factors on the composite D index. Overweight-obesity had intermediate priority and all other factors had much lower priority. Overweight-obesity, hypertension, low fruits and vegetables intake, smoking and insufficient Pap smear were the top five priorities based on a composite score of the three PHI that had cost and effectiveness excluded from their calculations (data not shown in tables).

## Discussion

To our knowledge, this is the first study to present a prioritization model for health intervention for Brazil urban areas and the majority of population. Our results show that hypertension, physical inactivity, blood pressure screening, diagnosed hypercholesterolemia, smoking and binge drinking are the leading burdens and have the most impact on health. It also shows overweight-obesity as an intermediate priority for prevention in Brazil. Indeed, our findings present a road map for developing and implementing prevention programs or for accelerating existing ones. These programs are crucial for a country with a free health system, and a growing and aging population.

The World Health Organization (WHO) included five of these factors among the top seven priorities for middle-income countries and top five priorities for high-income countries [22]. Moreover, the Brazilian Ministry of Health (MH) National Policy of Health Promotion, launched in 2006, targeted directly physical inactivity, smoking and excessive alcohol intake, while indirectly targeting overweight-obesity and hypercholesterolemia through “poor diet” among its five targets for health promoting strategies [23]. More recently, physical inactivity, smoking and overweight-obesity have been targeted with specific actions through the 2011–2022 Strategic Planning for Tackling NCD in Brazil [24].

Our findings of an increasing prevalence of overweight and obesity in urban areas have been previously reported. One research reported an increase in adult obesity from 10.8% to 13.5% between 2006 and 2009 [25]. Another reported a higher rate of obesity increase from 1989 and 2009 among poor individuals compared to non-poor [26]. Indeed, this means that large societal changes led to an imbalance between caloric intake and expenditure in the country. As a result, the country has launched a nationwide program to increase physical activity and improve diet, the Academias da Saude (i.e., Health Academies) [24]. This rise in obesity and overweight deserved further attention and proper management through prevention in Brazil. High obesity levels for long periods of time will make it a norm for the population and there will be fewer incentives for individuals to take action if obesity becomes acceptable.

MH goal is to expand Academias da Saude to 4,000 municipalities by 2011 [24]. Brazil’s promotion of physical activity started six years before Academias da Saude (2005) through MH annual funding to the 27 capital cities for local interventions to promote physical activity and health [27]. By 2010, this physical activity network already included 469 projects. This shows the commitment of MH and local health authorities to deal with the health priorities and receptivity by communities. Therefore, this effort should be supported as a means to share experience, and lessons should be adopted to ensure that success stories are copied.

Diagnosed hypertension, blood pressure screening and diagnosed hypercholesterolemia were among the top priorities. Hypertension and hypercholesterolemia-related cardiovascular morbidity and death are significant public health issues in Brazil. Though cardiovascular disease standardized mortality rates decreased in Brazil in the past two decades, an increase in cardiovascular deaths is expected in the next decades [28]. Since 2011, hypertension-related complications and deaths have been directly targeted with preventive actions by MH. In February, 2011, MH initiated an unprecedented strategy of offering free medication to control and reduce the burden of hypertension, diabetes and asthma: the Saude nao tem Preco program (i.e., Health is Priceless) program [29]. The Saude nao tem Preco distributes, free of charge, 11 medicines--six for hypertension and five for diabetes--to control blood pressure and diabetes. As of February, 2014, the program, active in 4,119 cities through a network of 30,136 pharmacies, has provided services to 6.6 million diabetic and 16.4 million hypertensive patients [30]. This program will no doubt reduce the burden of blood pressure and diabetes as long as patients are properly followed to ensure the medication is controlling their conditions. However, changes in health behaviors and proper clinical management of hypercholesterolemia as in the use of statins should also be promoted and such advices regularly given [31].

We found about 84% of deaths were attributable to hypertension, physical inactivity, smoking, overweight-obesity, and binge drinking in our study. Our report of the large death burden attributed to preventable factors such as smoking and alcohol have been reported previously [32,33]. In 2003, researchers estimated that 24,222 out of 177,543 total deaths in Brazil were attributable to smoking [32]. This figure is slightly higher than ours that includes only capitals. Indeed, this is also due to the declining smoking rates in the country. A 46% reduction of smoking rates between 1989 and 2010 is due to national policies, including taxation (1990), banning of advertising (1996), warning on packages, and smoke-free laws [34]. The government of Brazil signed, ratified and enforced the Framework Convention on Tobacco Control (FCTC) in 2003, 2005 and 2006, respectively [24]. The FCTC has really made an impact on tobacco smoking and a reduction has been seen since its implementation.

Our estimated alcohol attributable deaths are lower than previously reported in 2006 (22,927deaths compared to 23,608 deaths) [33]. Differences in estimation methodology, geography (i.e., only capitals), and year prevalence of alcohol intake and deaths calculated could explain this difference. Alcohol consumption is high in Brazil with men consuming nearly 20 liters of pure alcohol while women consumed 8.9 liters in 2010 [35]. In our study, binge drinking ranked high because of the excessive number of deaths attributed to alcohol, mainly due to traffic accidents, other accidents and violence. Indeed, traffic accidents are responsible for more than 150,000 injuries every year with 35,000 fatal outcomes, and an estimated cost of USD $14 billion per year [36]. However, recent policies have the potential to reduce the alcohol burden in Brazil. In a recent report by WHO, Brazil had implemented 10 out of 12 policies known to reduce excessive intake of alcohol [35]. In 2008, Brazil introduced a policy consisting of near zero tolerance on alcohol intake (legal BAC limit at 0.02 g/l) while driving [37]. Initial reports have shown a decline in alcohol burden manifested by reduction in hospital admissions, health care costs and deaths related to traffic accidents [38,39].

We found the highest health disparity based on education for insufficient Pap smear and mammography screening in our study. An estimated 52,680 new cases of female breast cancer and 17,540 new cases of cervical cancer were reported in 2012 [40]. Moreover, a recent review of breast cancer in Brazil revealed low awareness of breast cancer danger and low screening levels [41]. Indeed, early detection should be a priority for the country to avoid complications and increase the chance of a cure. Several studies in Brazil reported large disparities in Pap smear and mammography screening by education, race, and other socio-economic factors [42,43]. Therefore, examining the reasons for disparities will help in finding solutions to address the low screening in certain segments of the population. Moreover, it would allow the MH to target programs to those in need and maximize the utilization of available resources.

We found a significant and positive trend of diabetes in Brazil. Previous studies reported increases in the prevalence, hospitalization and deaths due to diabetes [1]. Another study estimated that the prevalence of diabetes increased by 20% from 2006 to 2010 [44]. Diabetes causes 278,778 years of potential life lost for every 100,000 people in Brazil, with an annual direct cost of USD $3.952 billion in 2000, and an estimated annual indirect cost of USD $18.6 billion [44]. The introduction of new guidelines for diabetes prevention, screening, diagnosis, initial evaluation, and basic treatment in 2006 by MH has improved the management of diabetes [45]. Moreover, medication has been freely available since 1971 through the public health services and at 10% of its market price through the Farmacia Popular (i.e., Popular Pharmacy) program since 2006 [46]. In 2011, the Saude nao tem Preco made metformin, glibenclamide, Human insulin, and NPH insulin freely available for all through the Farmacia Popular [47]. Indeed, the impact of these programs will unfold in the coming years as there is a lag between a program and outcome. However, these programs will reduce the burden of diabetes as long as patients are being monitored and make the necessary behavioral changes.

All six priority factors we report have effective interventions to reduce their prevalence between 8% and 35% with per capita costs between $10 and $42 per person reached [14]. A recent study estimated that 5% reduction in the mean BMI in Brazil could reduce obesity and prevent 2.1 million prevalent cases of hypertension by 2050 [48].

Our study has some limitations. Risk and related factors for infectious and other diseases in Brazil are not available. Though the study focused on the combined data of 27 capital cities to generate the health priorities for urban Brazil, priorities for each of the nearly 4000 municipalities in Brazil including rural areas may be very different.

A major limitation of the PHI methodology is its dependence on the quality of the surveillance data as in the VIGITEL and SIM. VIGITEL’s main limitations are the differential telephone coverage and survey nonresponse that may result in significant differences of the studied variables between individuals with and without a telephone line, and respondents and non-respondents [49,50]. But, a recent study reported that 15 out of 18 indicators of VIGITEL were reproducible and valid [51]. Furthermore, the VIGITEL questionnaire only allows for creation of a weekly frequency of consumption of fruits and vegetables, while the recommended measure based on risk of chronic diseases is five times a day [52]. Nevertheless, our categorization was sufficient to differentiate cities as high, mid-high, middle and low consumption of fruits and vegetables (data not shown in tables). SIM’s main limitation is the completeness of death registration as expressed by the reporting of high numbers of deaths with undetermined causes, mainly in the North-east of Brazil [53]. However, recent studies show improvement in death reporting with 80% of municipalities providing valid statistics [54]. In addition, the weights used to generate factors prevalence estimates may render population attributable risk estimates biased compared to other methods [55,56]. Yet, PHI remained stable (i.e., its values did not change more than 10%) after simulations with 10% change in the value of one or more health indicators. Finally, the estimate of the number of attributable deaths used in PHI may be biased [57].

A major strength of PHI is the ability to incorporate other criteria and indicators. For example, the state of Missouri interactive PHI (Priorities MICA) available in the internet since 2002, adds other indicators (e.g., DALY), and its dashboard allows users to incorporate a criterion of community support for a public health issue [12,13]. PHI normalizes and harmonizes hundreds of statistical calculations from multiple factors and diseases indicators simultaneously, thus facilitating the prioritization process. PHI may allow prioritization for population groups defined by region, age, race/ethnicity, or sex group as well as identify specific indicator, disease or factor weighing on the ranking of the index.

## Conclusions

The top six priority factors for NCD in Brazil are hypertension, physical inactivity, blood pressure screening, hypercholesterolemia, smoking and binge drinking. While, as of 2011, these six factors have been addressed in Brazil with health promotion and prevention strategies, more resources and effective strategies are needed to address these factors and sustain gains. We provide a model for the MH in Brazil to set priorities for intervention programs. We believe this model is of great value and could be used to monitor progress and evaluate interventions in Brazil and countries with similar surveillance systems. Moreover, the PHI model is simple to implement but sophisticated in analyses, and it allows for health officials to apply it in their own settings (e.g., locally or nationally).

## Additional files

Additional file 1:
**Risk Factors for Chronic Diseases (English) – Vigitel 2010.**
Additional file 2:
**Disease Groups and ICD (9 and 10) Used for Calculating Attributable Deaths.**
