# Supplementary material for: A priority health index identifies the top six priority risk and related factors for non-communicable diseases in Brazilian cities
Source: BMC Public Health. 2015 May 1;15:443. doi: 10.1186/s12889-015-1787-1 (PMC4425866; doi:10.1186/s12889-015-1787-1)
Supplement: Additional file 1: — Risk Factors for Chronic Diseases (English) – Vigitel 2010. [file 12889_2015_1787_MOESM1_ESM.docx]

**Appendix 1. Risk Factors for Chronic Diseases (English) – Vigitel 2010**

| Risk Factor | Definition |
| --- | --- |
| Current Smoking | **Percentage of smokers:** number of smokers / number of individuals interviewed. Smoking was considered the individual who responded positively to the question "Do you smoke?", Regardless of the number of cigarettes, the frequency and duration of smoking. |
| Physical inactivity | **Percentage of physically inactive adults:** number of physically inactive individuals / number of individuals interviewed. Was considered physically inactive adult who did not practice any physical activity during leisure time in the last three months, and not made ​​physical exertion at work, not moved to work on foot or by bicycle, and was not responsible for heavy cleaning of your home, as combination of indicators below *. |
| Overweight-Obesity | **Percentage of adults who are overweight:** the number of overweight individuals / number of individuals interviewed. Was considered overweight individuals with Body Mass Index (BMI) ≥ 25 kg/m2 (WHO, 2002), calculated as weight in kilograms divided by the square of height, both self-reported, as the questions: "Do you know your weight (even approximately)? "," Do you know your height? " Percentage of obese adults: number of obese individuals / number of individuals interviewed. Was considered obese individuals with body mass index (BMI) ≥ 30 kg/m2 (WHO, 2002), calculated as weight in kilograms divided by the square of height, both self-reported, according to the questions: " Do you know your weight (even approximately)? "," Do you know your height? " |
| Eating <5 fruits/vegetables per week | **Percentage of adults who consume fruits and vegetables regularly:** number of individuals with regular consumption of fruits and vegetables / number of individuals interviewed. Was considered regular consumption of fruits and vegetables, consumption of five or more days per week of fruits and vegetables, estimated from responses to the questions: "How many days of the week you usually eat fruit?" and "how many days of the week you usually take natural fruit juice?" and "how many days of the week you usually eat at least one type of vegetable or vegetable (lettuce, tomato, cabbage, carrots, chayote, eggplant, zucchini - not worth potatoes, cassava or yam)? " |
| Abusive drinking of Alcohol (Binge Drinking) | **Percentage of adults who consumed alcohol abusively (Binge Drinking):** number of adults who consume alcohol abusively / number of respondents. Was considered excessive consumption of alcoholic drinks more than five drinks (for men) or four doses (woman) on a single occasion at least once in the last 30 days. A consumer was considered abusive if respondents answer yes to the question "In the last 30 days, have you consumed more than 5 doses * of alcohol on a single occasion?" For men or "In the last 30 days, have you consumed more than 4 doses * of alcohol on a single occasion?” For women. An alcoholic drink corresponds to a can of beer, a glass of wine or a shot of rum, whiskey or other distilled spirits.  Note: The alcohol abuse measure is comparable starting on the 2007 survey. In 2009, the limit has to be four or more drinks for women and five or more drinks for men. |
| Insufficient Papanicolau Screening - longer than 3 years | **Percentage of women (25-59 years) who had not underwent Pap test in the last three years:** women between 25 and 59 years of age who had not underwent Pap test in the last three years / number of women aged 25 to 59 years old interviewed, in answer to the question: " How long has it been since you took a Pap test? |
| Insufficient Mammography Screening - longer than 2 years | **Percentage of women (50-69 years) who had not underwent mammography in the last two years:** women between 50 and 69 years of age who had not underwent mammography in the last two years / number of women between 50 and 69 years old who were interviewed, as response to question: " How long has it been since you took a mammogram?" |
| Insufficient Blood pressure screening- longer than 1 year | **Percentage of adults who had underwent measure of blood pressure in the last year:** number of adults who had not underwent measure of blood pressure in the last year / number of adults interviewed, as answer to the question:" How long has it been since you took a measure of Blood pressure? " |
| Insufficient glucose screening - longer than 2 years | **Percentage of adults who underwent blood glucose measurement in the last two years:** number of adults who underwent blood glucose measurement in the last two years / number of adults interviewed, as answer to the question: "How long has it been since you took measure of glucose in the blood? " |
| Diagnosed with hypercholesterolemia | **Percentage of adults who reported a diagnosis of dyslipidemia:** number of individuals who reported a diagnosis of dyslipidemia / number of individuals interviewed, regarding the positive response to the question: "Has any doctor ever told you that you have dyslipidemia?" |
| Diagnosed with hypertension | **Percentage of adults who reported physician-diagnosed hypertension:** number of individuals who reported physician-diagnosed hypertension / number of individuals interviewed as an affirmative answer to the question: "Has any doctor ever told you that you have high blood pressure?” |
| Diagnosed with diabetes | **Percentage of adults who reported physician-diagnosed diabetes:** number of individuals who reported physician-diagnosed diabetes / number of individuals interviewed, regarding the positive response to the question: "Has any doctor ever told you that you have diabetes?" |

* For better understanding of the definition of the indicator " Physical Inactivity " it is necessary to explain the four forms of " physical activity " :
1) Percentage of adults who engage in sufficient physical activity in leisure: the number of individuals who engage in sufficient physical activity in leisure / number of individuals interviewed . It was considered active in the adult leisure activity practiced mild to moderate (walking, treadmill walking , weight training , aerobics , general gymnastics , swimming, martial arts and fighting, cycling , volleyball or other ) for at least 30 minutes daily in 5 or more days per week or vigorous activity (running, treadmill running , aerobics , soccer , basketball or tennis ) for at least 20 minutes daily for 3 or more days of the week , according to the questions : " in recent three months, did you practice some type of exercise or sport ? "What is the main type of exercise or sport that you practiced? ", "Do you practice the exercise at least once a week? "How many days per week do you usually practice exercise or sport?" and "when you practice exercise or sport , how long does this activity last ? "
2) Percentage of physically active adults in employment: number of physically active individuals occupational / number of individuals interviewed. It was considered physically active in occupational an adult who performs physical exertion at work, positive response to the questions: "In the last three months, did you work?" And "when you work out, do you walk quite a lot?" or "In your work, do you carry weight or exercise other strenuous activity? "
3) Percentage of physically active adults in transport / shift: number of physically active transport / number of individuals interviewed. It was considered physically active in transport the adult who traveled to work on foot or by bicycle, on a positive response to the questions: "In the last three months, did you work?" And "Do you usually walk or bike to work from home?" and 20 minutes or more to the question "How long do you spend to get to and from work (walk or bike)?"
4) Percentage of physically active adults in the household: number of physically active household / number of individuals interviewed. Was considered physically active in the household the adult who is responsible for heavy cleaning of your home considering the answer myself the question "Who usually does the cleaning of your house?" Or "Who gets the heaviest part of housecleaning, when you have help?"
