# Supplementary material for: A priority health index identifies the top six priority risk and related factors for non-communicable diseases in Brazilian cities
Source: BMC Public Health. 2015 May 1;15:443. doi: 10.1186/s12889-015-1787-1 (PMC4425866; doi:10.1186/s12889-015-1787-1)
Supplement: Additional file 2: — Disease Groups and ICD (9 and 10) Used for Calculating Attributable Deaths. [file 12889_2015_1787_MOESM2_ESM.docx]

Appendix 2 – Disease Groups and ICD (9 and 10) Used for Calculating Attributable Deaths

| **Causes of Death** | **ICD-9** | **ICD-10** |
| --- | --- | --- |
| tuberculosis | 010-018 | A15- A19 |
| Lung Cancer (trachea, bronchus and lung | 162-1629 | C33-C349 |
| Cancer of the Bladder | 188-1889 | C670-C679 |
| Breast cancer | 174-1759 | C500-C509 |
| Bowel cancer | 153 | C18 |
| Cancer Colorectal | 153- 154 | C18- C20 |
| Digestive Cancer (colorectal, stomach and esophagus | 150,151,153,154 | C15-C21 |
| Malignant neoplasm of kidney |  | C64 |
| Malignant neoplasm of the Kidney and Renal Pelvis | 1890-1891 | C64-C65 |
| Malignant neoplasm of lip, oral cavity and pharynx | 140-1499 | C00-C14 |
| Esophageal cancer | 150-1509 | C15 |
| Stomach Cancer | 151-1519 | C16 |
| Cancer of the Pancreas | 157-1579 | C25 |
| cervical cancer |  | C53- C54 |
| Malignant neoplasm of uterus |  | C54 |
| Malignant neoplasm of cervix and uterus | 180-1809 | C53- C54 |
| Acute myeloid leukemia | 2050 | C920 |
| Larynx cancer | 161 | C32 |
| Cancer of the Liver | 155-1552 | C22-C229 |
| Cancer of the mouth and pharynx | 140-1499 | C04, C10-C13 |
| Prostate cancer | 185-1859 | C61 |
| Non Hodgkin Lymphoma | 200, 202 | C82-C85, C96 |
| Selected other cancer | 210-239 | D00-D48 |
| Circulatory system | 390- 4599 | I00-I99 |
| Cardiac arrhythmia | 427 | I47-I49 |
| Hemorrhagic infarction | 431-432 | I61-I62 |
| aortic Aneurysm |  | I71 |
| atherosclerosis | 440 | I70 |
| Ischemic Heart Disease | 410-4149 | I200-I259 |
| Other Heart Disease |  | I30-I50 |
| Cerebrovascular diseases | 430-438 | I60.0-I69.8 |
| Hypertensive diseases | 401-4059 | I10-I13 |
| Cerebral Infarction - Myocardial Ischemic | 434 | I63 (no I637) |
| Other Arterial Disease |  | I73- I74, I77- I78 |
| Other cardiovascular diseases | 390-398, 415-429, 440-459 | I00-I09, I26-I52, I70-I79 |
| Pneumonia and Influenza | 480-488 | J100-J189 |
| Other acute lower respiratory infections and asthma | 466 ,493 | J20-J22, J45 |
| Chronic Obstructive Pulmonary Disease (COPD) | 493- 496 | J44 |
| diabetes mellitus | 250-2509 | E10-E14 |
| Chronic hepatitis | 57140 | K73 |
| esophageal varices | 4560-4562 | I85 |
| Fetal alcohol syndrome | 76071 | Q860 |
| Degeneration of nervous system due to alcohol | 3575, 4255, 5353 | G312 |
| Fetus and newborn affected by maternal use of alcohol |  | P043 |
| Alcohol-induced chronic pancreatitis |  | K860 |
| Bronchitis, Emphysema | 490-492 | J40- J43 |
| Gastro-esophageal laceration hemorrhagic syndrome- | 530.7 | K226 |
| epilepsy | 345 | G40 |
| acute pancreatitis | 577 | K85 |
| Acute and chronic pancreatitis | 5770-5771 | K85, K860-K861 |
| Alcohol abuse |  | F10-F109 |
| alcoholic cardiomyopathy | 4255 | I426 |
| Mental and behavioral disorders due to use of alcohol dependence syndrome |  | F102 |
| alcoholic polyneuropathy | 3575 | G621 |
| Alcohol-induced chronic pancreatitis |  | K860 |
| alcoholic gastritis | 535.3 | K292 |
| Alcoholic liver disease | 571.0-571.3 | K700-K709 |
| alcoholic myopathy | 359.4 | G721 |
| alcoholic psychosis |  |  |
| Cirrhosis of the liver | 571 | K703, K717, K74 |
| Prematurity, low birth weight | 765 | P07 |
| Portal hypertension | 572.3 | K766 |
| psoriasis | 696 | L40 |
| miscarriage | 634 | O03 |
| Stroke, not specified as hemorrhage or infarction | 436 | I64 |
| supraventricular tachycardia | 42789 | I471 |
| suicide | E950-E959 | X60-X84, Y10-Y34 (no Y33.9) |
| Accidental poisoning by and exposure to alcohol |  | X45 |
| Accidental drowning and submersion | E910 | W65-W74 |
| Intentional self-harm by smoke, fire and flames | E890-E899 | X76 |
| Injury by firearms | E9550-E9554 | X73-X74 |
| hypothermia | E9010, E9018, E9019 | X31 |
| Motor Vehicle Traffic – Transport related Accident |  | V01- V89 |
| Falls, homicide, suicide, and death from other lesions |  | W00-Y34 |
